# The Motivational Level of Performance Swimmers and Its Impact on the Risk of Sports Dropout

**DOI:** 10.3390/sports13040125

**Published:** 2025-04-17

**Authors:** Valentina Brat, Aura Bota, Georgeta Mitrache, Silvia Teodorescu

**Affiliations:** Faculty of Physical Education and Sport, National University of Physical Education and Sports, 060057 Bucharest, Romania; id.tina@yahoo.com (V.B.); georgeta.mitrache@unefs.ro (G.M.); silvia.teodorescu@unefs.ro (S.T.)

**Keywords:** sports motivation, adolescent swimmers, dropout prevention

## Abstract

Background: Motivation is a crucial factor in maintaining athletic performance and preventing dropout among competitive athletes. This process is influenced by both physical and psychosocial factors, which interact and shape decision—making regarding continued participation or withdrawal from sports. Aim: This study examines the motivational level of competitive swimmers in Romania, related to the dropout perspective. Methods: In order to conduct the research, the AMI (Achievement Motivation Inventory)—a validated psychometric tool was used in two distinct phases, conducted six months apart. The study included N = 20 swimmers, finalists and medalists in national swimming competitions. The intervention consisted of motivational coaching, personalized training plans, and the development of mental skills. Results: The results highlighted significant improvements in dimensions such as success confidence, compensatory effort, and goal-setting, indicating the positive effects of the intervention on athletes’ motivation. Inferential analysis using Student *t*-test confirmed significant differences between the initial and final assessments, for eagerness to learn (*p* = 0.035), status orientation (*p* = 0.03) and the Wilcoxon test revealed significant difference for general motivational index (*p* = 0.020). Conclusions: The findings underscore the importance of psychological approaches in training high-performance athletes, showing that maintaining motivation and clarifying goals are essential factors in preventing sports dropout. The conclusions of this research can serve as a foundation for developing coaching strategies aimed at supporting continuity in performance swimming and reducing the dropout rate among competitive swimmers. Our findings confirm similar studies emphasizing the role of the training patterns and coach influence not just on the performing athlete, but also on his psychosocial individual development.

## 1. Introduction

Sports dropout is a challenging topic in high-performance sports, especially in disciplines such as swimming, which require rigorous training and strong mental resilience. Sports dropout refers to the voluntary, conscious, and premature decision of athletes to discontinue systematic training and competition participation before reaching their full potential. Some experts suggest that the reasons for participation and withdrawal from sports may not be directly connected and emphasize the importance of understanding the processes young athletes go through before quitting sports. These processes are influenced by both physical factors (maturation level, training patterns) and psychosocial factors (coaches, parents, siblings, peers), which interact and shape decision-making regarding continued participation or withdrawal from sports [1].

The decentering concept, mentioned by Diotaiuti, Valente, Corrado & Mancone (2023) is like a mental technique, which helps athletes to step back from negative emotions and convert stressful situations in opportunities to further develop their sport career, alleviating the dropout tendency [2].

Existing studies highlight that motivation plays a central role in developing athletic skills and achieving competitive goals, being influenced by factors such as the training environment, social support, personal objectives, and the perception of success [3,4]. Authors like Rocchi & al (2020) analyzed athletes’ perceptions of coaches’ behaviors and their motivation, as predictors of future sport results [5].

To date, most research on sports withdrawal has relied on two main theoretical frameworks: achievement goal theory and self-determination theory [6]. Researchers have, thus, sought to analyze coping strategies and achievement motivation dimensions among elite athletes, considering these variables as essential resources for optimal performance in sports. Achievement motivation not only aids in reaching higher goals but also in sustaining long-term engagement in sports [7], particularly in light of the changes observed among adolescents and experienced athletes who drop out of competitive sports for various reasons. In this context, sports dropout can be seen as a maladaptive behavior resulting from low self-determination [8], influenced by interpersonal relationships and specific circumstances [3]. These two theories provide a perspective on children’s and adolescents’ motivation to engage, persist, or withdraw from sports.

In individual sports such as swimming, athletic motivation is frequently assessed and monitored to prevent declining interest and potential dropout issues often reported in international endurance sports research [9,10].

Competitive swimming requires constant commitment and a high level of self-discipline. Therefore, young athletes often face challenges in balancing academic demands with intense training schedules, which can lead to demotivation and premature dropout. The study by Fraser-Thomas et al. (2008) highlights factors contributing to sports dropout, including lack of time, performance stagnation, health issues, and the perception of an overly demanding training environment. In this context, motivational testing becomes a crucial tool for identifying risk factors and implementing timely interventions to prevent sports dropout [11].

This phenomenon in high-performance sports poses a major challenge for coaches and sports clubs, with long-term implications for athletes’ physical and psychological well-being. 

The findings of Pissaniello A. et al. (2024), in Italy, reinforce the need for a balanced and supportive sports framework that prioritizes young athletes’ well-being (age 8–13), ensuring they can fully experience the physical, psychological, and social benefits of sports participation [12].

Interventions aimed at increasing motivation can have a significant impact on athletes’ performance and their decision to continue practicing high-performance sports. A positive training environment focused on personal development and long-term goal setting can prevent burnout and encourage athletes to maintain their sports careers. The study of Beaudoin C. et al. (2015) underscores the importance of the Long-Term Athlete Development (LTAD) model as a foundational framework for Canadian sport, with widespread adoption among sport governing bodies [13]. Overall, while the LTAD model presents a valuable approach to long-term athlete development, its full integration requires stronger institutional support, clearer practical applications, and ongoing research to validate its effectiveness across all sports disciplines.

The present study is particularly relevant for Romania, where the phenomenon of sports dropout among performance swimmers represents a major challenge for coaches and sports clubs.

## 2. Materials and Methods

The purpose of this research is to analyze the motivational levels of competitive swimmers in Romania and to assess the impact of a personalized coaching intervention on sports motivation using the Adolescent Motivation for Interpersonal and Achievement Scale test (AMI), adapted for Romania under AMI license OL-00011862/2024-01-30.

**Hypothesis.**  A structured psychological intervention on the performance swimmers within the precompetitive period will help enhance the motivational dimensions revealed in the AMI scale test.

The study seeks to identify motivational changes recorded across two distinct testing phases, conducted six months apart, as well as to highlight the factors that contribute to maintaining athletic performance and preventing sports dropout. This research aims to provide practical recommendations for coaches and sports specialists by developing personalized coaching programs designed to support long-term engagement in sports and reduce dropout rates among adolescent swimmers.

At the core of our intervention is the G.R.O.W. model (Goal, Reality, Options, and Will), which guided athletes in goal setting and action planning. Through brainstorming sessions and individual discussions, athletes were encouraged to explore their options, analyze their strengths, and develop personalized strategies to enhance their performance. In the final stage of the model, athletes committed to implementing their action plans, with ongoing support from their coaches throughout the process.

The AMI is a validated psychometric tool used to assess athletes’ motivation. In this study, the initial AMI testing was conducted in January 2024 via the Test Central platform. The participant group included 20 competitive swimmers, consisting of 10 female and 10 male athletes, aged 16 to 22 years, all of whom were finalists or medalists in national swimming competitions. The research did not aim at comparing the gender oriented motivational dimensions, but intended to emphasize the progress for each group, after the experimental period.

The final testing took place in July 2024, using the same platform and methodology.

These findings highlight the diversity of factors influencing athletes’ decisions to withdraw from competitive sports, emphasizing personal, professional, performance-related.

During the research, it was observed that some athletes withdrew from the project or even from competitive swimming altogether. Of the 20 participants who initially took the AMI test, five athletes (two boys and two girls) later dropped out. The reasons cited (Table 1) align with findings from other studies on the determinants of sports dropout among young athletes [14]. The findings of the systematic review conducted by Back J. et al. (2022) highlight the crucial role of motivation and sport experience in preventing dropout among adolescent team sport athletes [15]. The results emphasize the need for sports organizations, coaches, and parents to foster a high-quality motivational climate that satisfies athletes’ psychological needs and encourages long-term commitment. The strongest predictors of dropout were related to self-determined motivation, attitudes, and social norms, reinforcing the importance of autonomy-supportive environments. Additionally, the study identifies a gap in prospective research on dropout determinants, underscoring the necessity for further investigations. Implementing educational programs for coaches focused on autonomy-supportive coaching strategies can significantly enhance athlete retention and mitigate dropout risks. 

A systematic review by Monteiro et al. (2017) stated that regardless of the research design of the analyzed studies, the main reasons related to dropout in swimming include: conflicts with coaches’ failure in competence improvement, parents’ pressure, lack of fun, boredom, etc [16]. Thus, preventing dropout has to address its determinants, in the long run.

Although objective performance is important, and the ultimate goal is to improve athletic results, the primary objective of our research was to evaluate the psychological components of sports dropout.

The low number of athletes participating in the AMI testing can be explained by the fact that competitive swimming in Romania is a niche sport, with a limited number of athletes reaching a high competitive level. As presented in Table 2, there is a certain dynamics of the number of participants in the *National Swimming Championships for Seniors*, *Youth*, *and Juniors*, but only a small fraction make it to the finals and win medals.

Under these circumstances, the study group represents the top tier of this age category, consisting exclusively of finalists and medalists at national competitions. Therefore, the small number of participants is justified by the fact that few athletes meet the performance profile targeted by the research, which adds relevance and specificity to the obtained results. Additionally, these athletes exhibit a higher level of motivation, focused on performance, and follow a relatively uniform athletic lifestyle, characterized by a comparable training schedule and educational path. By analyzing this specific group, we aimed to gain deeper insights into the factors contributing to their success, as well as the challenges they face.

The AMI test (extended version) includes 170 items, with responses recorded on a 7-point Likert scale. The results are grouped into 17 structural scales, and a global motivational index is calculated. The test administration time varies from 10 to 30 min (TestCentral—D&D Consultants Grup—O.S. Organizzazioni Speciali Romania).

The standard, brut and stanine score, raw score, and stanine score are terms used in the evaluation and interpretation of AMI test results and serve as psychological measurement tools. These terms are employed to compare and interpret an athlete’s individual performance against a given norm or reference distribution.

Based on the analyzed AMI test data, we implemented coaching techniques to improve the athletic training plan. The AMI test battery specifies that perseverance and athletic performance variables do not have a direct significant relationship, but we can still adjust training sessions to maximize athletic potential.

Awareness is essential in any athletic activity, as it provides control over reality and allows athletes to understand and improve their performance. In competitive sports, kinesthetic awareness—the ability to perceive one’s own body movements—is crucial. It helps athletes detect and quickly correct inefficiencies in their movements, leading to smoother and more efficient technical execution.

In order to enhance motivation, increase awareness of the training process, and improve relationships among teammates and with the coach, we evaluated and implemented the following strategies in the 2024 athletic training plan (Appendix A, Table A1b).

### 2.1. Motivation and SMART Goals (Mesostructure 7, January 22–March 31)

During this period, we implemented SMART goals (Specific, Measurable, Achievable, Relevant, and Time-bound) on short, medium, and long-term levels for each athlete, based on previous progress and individual objectives. This phase began immediately after the first AMI test in January, providing each athlete with clarity and direction.

The standard, brut and stanine score were essential tools for tracking development.

### 2.2. Constructive Feedback and Self-Assessment (Mesostructure 8, April 1–April 14)

Providing continuous and constructive feedback became central. After each major training session, athletes received personalized feedback focused on individual progress. Additionally, at the end of each mesostructure, we organized group reflection sessions, where athletes analyzed their performances and set new objectives for the upcoming period.

### 2.3. Awareness and Mental Visualization (Mesostructures 7 and 8)

To improve focus and technique, we introduced mental visualization sessions once per week. During these sessions, athletes visualized the perfect execution of swimming techniques, race progressions, and even the moment of victory.

We also introduced mindfulness training or short meditation sessions after important training sessions to manage stress and enhance concentration.

### 2.4. Team Building and Communication with the Coach (Mesostructure 7, January 22–March 31)

Team communication and relationships are essential for long-term success. We organized monthly team-building activities, such as trips or team games, to strengthen bonds among athletes. Additionally, we encouraged open communication between athletes and coaches through periodic discussion sessions, where athletes could give and receive feedback in a non-judgmental environment.

### 2.5. G.R.O.W. Model Integration

Team communication and relationships are essential for long-term success. We organized monthly team-building activities, such as trips or team games, to strengthen bonds among athletes. Additionally, we encouraged open communication between athletes and coaches through periodic discussion sessions, where athletes could give and receive feedback in a non-judgmental environment.

We also integrated the G.R.O.W. Model (Goal, Reality, Options, and Will), which helped athletes clarify their current situation, define their objectives, identify obstacles, and develop strategies to overcome them. This model was applied at the beginning of the preparation period and before competitions, enhancing confidence and performance.

By implementing these strategies in Macrostructure II, we aimed to provide each athlete with technical, psychological, and relational support, preventing dropout and improving both individual and team performance.

The interpretation of the AMI profile, which includes the analysis of scores for each scale, provides a detailed and coherent description of individual motivation for performance. This interpretation allows for the identification of intra-individual differences and specific aspects of performance motivation. Individuals with high scores on the general motivational index are often determined and motivated, displaying perseverance, a willingness to exert effort, and a strong belief that their own effort and abilities generate the desired results. These individuals may be confident in their abilities and personal success, or they may experience a fear of failure—both situations motivating them to put in additional effort.

For the statistical processing of the study data, the software IBM SPSS Statistics for Windows, Version 29.0 (Armonk, NY, USA: IBM Corp.) was used. Nominal data were presented as absolute frequency and percentage, while continuous variables were expressed through mean values, standard deviation, minimum, and maximum values.

In order to compare two sets of dependent or paired data, we used the Wilcoxon test, a nonparametric test, since the data did not meet the normality assumptions required for the paired-sample *t*-test. The Wilcoxon test is typically applied when two measurements are taken from the same subjects (e.g., before and after implementing an intervention plan) to assess whether a significant difference exists between the two measurements.

For comparing the means of parameters between groups, the Kruskal–Wallis H test was used, given that the variables exhibited a non-Gaussian distribution. A statistical significance coefficient of *p* < 0.05 was considered valid.

## 3. Results

We analyzed the descriptive and statistical results from two testing sessions to highlight significant differences in the scales, separating the two genders, female and male, by first examining the standard score. These are normalized scores with a mean of 100 and a standard deviation of 10, obtained by standardizing the raw scores. Standardized scores facilitate easier comparisons between different datasets and with other samples.

An increase in self-confidence, measured through the success security item (from 98.52 to 105.38), indicates an improvement in athletes’ confidence in their ability to achieve positive results, which can lead to enhanced competitive performance (Table 3 and Table 4).

An increase in the eagerness to learn (from 101.97 to 108.30) suggests a heightened interest in personal development and technical skill improvement—an essential factor in high-performance sports.

The clarification of personal goals, reflected by the goal-setting score (from 99.41 to 107.13), represents a key factor in maintaining long-term motivation.

Perseverance, fearlessness, flexibility, and dominance remained relatively constant, indicating the need for different strategies to stimulate these aspects. These traits may be harder to influence in the short term and are often affected by external factors such as competition-related stress, family environment, and peer relationships.

**Figure 1:**
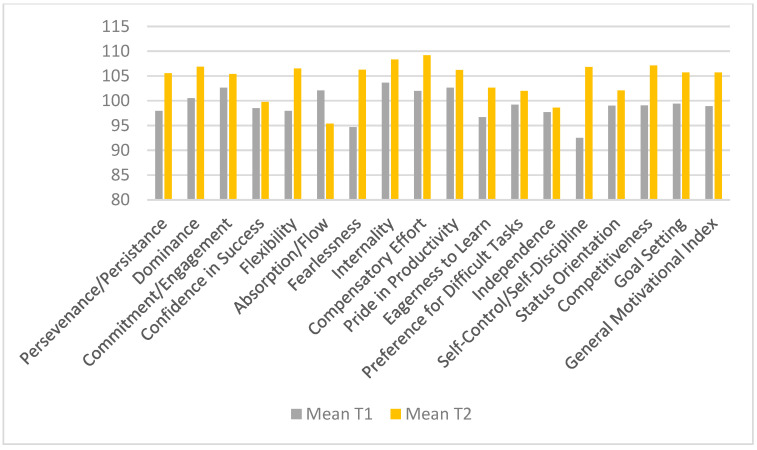
Descriptive analysis of both testings—T1 and T2 results based on the overall mean score.

Results highlight the positive effects of the intervention and provide a foundation for optimizing future strategies (Figure 1).

The Shapiro–Wilk test checks whether a variable follows a normal distribution. A *p*-value < 0.05 suggests that the variable does not meet the normality assumptions. Levene’s Test for Homogeneity of Variance checks if different groups have equal variances (a key assumption in ANOVA and other parametric tests). A *p*-value < 0.05 illustrates significant difference in variances and the assumption is not met.

Indicators that do not meet the assumption of normality are as follows: Pride in Productivity (W = 0.867, *p* < 0.001), Independence (W = 0.94, *p* = 0.05), right on the borderline and General Motivational Index (W = 0.937, *p* = 0.04).

For all variables that meet the assumption of normality and homogeneity of variance parametric tests (e.g., *t*-test, ANOVA, Regression, etc.) are recommended. For the variables that do not meet these criteria (Pride in Productivity *p* = 0.001 and General Motivational Index *p* = 0.04), non-parametric tests are recommended (Wilcoxon rank, Kruskal–Wallis)

Results (Table 5 and Table 6) highlight the positive effects of the intervention and provide a foundation for optimizing future strategies.

To examine whether there are statistically significant differences between two independent groups on various psychological/motivational indicators, the criteria to be met are *p*-value < 0.05 → statistically significant difference between groups and Cohen’s d → effect size, i.e., the practical importance of the difference: 0.2 = small, 0.5 = medium, 0.8 = large.

Table 6 presents Student *t*-test for variables with normal distribution and the Wilcoxon test for variable with non-normal distribution. The results emphasize that only three indicators show both statistical significance (*p* < 0.05) and moderate-to-strong effect sizes, indicating real and meaningful group differences: Desire to Learn (*p* = 0.03, d = −0.7345) has a medium-to-large Cohen effect, meaning a statistically and practically relevant difference, Status Orientation (*p* = 0.030, d = −0.7588) has a medium-to-large, meaning significant and impactful difference in scores, and General Motivational Index (*p* = 0.02 d = −0.62) has a medium-to-large group means differ significantly. The overall increase in motivation is significant, indicating the success of the intervention.

While not strictly significant, there are variables that show moderate effect sizes and should not be dismissed, especially in exploratory or applied research contexts. These variables include Self-Control/Self-Discipline (*p* = 0.080, d = −0.604), which shows a moderate effect; worth investigating further is the Goal Setting (*p* = 0.085, d = −0.596), which shows close to significance; moderate impact.

The remaining indicators (e.g., Perseverance, Dominance, Absorption, Fearlessness, etc.) did not yield significant *p*-values (*p* > 0.10) and have small or negligible effect sizes, suggesting group means are statistically and practically similar.

### 3.1. Significant Increases After the Intervention in Male Subjects (Table 7, Figure 2)

- Dominance: the increase between the two tests (103.91 → 108.82) indicates a significant improvement in self-confidence and the athletes’ willingness to take initiative in difficult situations (*p* = 0.05). This progress suggests that the intervention contributed to developing leadership traits and enhancing athletes’ ability to make effective decisions under pressure.
- Confidence in Success: the improvement in confidence in achieving set goals (105.43 → 111.17, *p* = 0.05) may be attributed to psychological interventions that emphasized the importance of positive thinking and effective goal-setting strategies.
- Compensatory Effort: the significant increase in compensatory effort (*p* = 0.012) indicates that athletes are more willing to put in extra effort to overcome obstacles and challenges. This improvement is essential in high-performance sports, where resilience and the ability to cope with challenges are critical.
- Goal Setting: the significant improvement in goal setting (*p* = 0.025) indicates that athletes set clearer and more ambitious goals. This also includes the ability to develop effective strategies to achieve these goals. The progress may be attributed to interventions that emphasized the importance of planning and setting short-, medium-, and long-term objectives.
- Pride in Productivity: *p* = 0.021; the significant improvement in performance pride reflects increased satisfaction with their achievements. This can lead to stronger intrinsic motivation and a greater desire to continue progressing.
- Eagerness to Learn: *p* = 0.017; the increase in the desire to learn indicates greater openness among athletes to improve their skills and learn from new experiences, as well as their receptiveness to feedback.
- Status Orientation: *p* = 0.017; status orientation has significantly increased, indicating that athletes are more motivated to improve their position and recognition within the group or competitions.
- General Motivational Index: *p* = 0.012; athletes exhibit a stronger motivation to achieve high performance and remain engaged in their sport. This aspect is crucial for maintaining long-term commitment and reducing the risk of sports dropout.

**Table 7:** Descriptive Analysis—Male Group (Initial and Final Testing).

Indicators	N		Mean		Standard Deviation		Minimum		Maximum	
Dimensions	I	F	I	F	I	F	I	F	I	F
Perseverance/Persistence	10	8	100.69	98.63	14.62	14.23	82.34	82.34	122.23	119.57
Dominance	10	8	103.90	108.81	10.91	10.80	92.48	100.20	123.35	127.98
Commitment/Engagement	10	8	109.91	110.51	9.28	12.75	96.65	95.19	127.26	128.72
Confidence in Success	10	8	105.43	111.16	10.42	12.81	91.75	95.94	122.47	129.46
Flexibility	10	8	97.76	104.07	9.819	6.869	86.60	91.91	113.19	114.96
Absorption/Flow	10	8	105.00	108.52	7.909	14.54	95.57	88.73	116.09	128.40
Fearlessness	10	8	95.91	97.108	15.49	16.60	74.58	85.66	125.83	131.37
Internality	10	8	104.12	106.76	12.09	11.30	85.25	91.83	123.33	123.33
Compensatory Effort	10	8	106.81	114.08	12.00	9.294	88.05	107.11	126.17	129.11
Pride in Productivity	10	8	106.74	112.92	5.901	3.075	96.73	109.25	114.26	116.76
Eagerness to Learn	10	8	98.30	112.15	16.29	13.29	68.31	97.15	122.15	133.69
Preference for Difficult Tasks	10	8	104.19	105.60	17.03	16.08	85.04	81.21	134.85	131.02
Independence	10	8	100.91	105.22	11.39	10.56	86.05	92.80	118.14	123.21
Self-Control/Self-Discipline	10	8	95.10	101.05	8.533	14.42	83.86	82.42	109.80	128.53
Status Orientation	10	8	102.28	110.95	7.963	8.66	92.38	102.54	115.25	122.87
Competitiveness	10	8	101.37	105.33	14.77	13.75	75.88	89.89	124.90	127.70
Goal Setting	10	8	103.46	111.53	10.91	14.15	87.75	92.61	118.54	134.75
General Motivational Index	10	8	103.62	110.37	11.15	13.24	90.96	95.93	126.03	134.11

**Figure 2:**
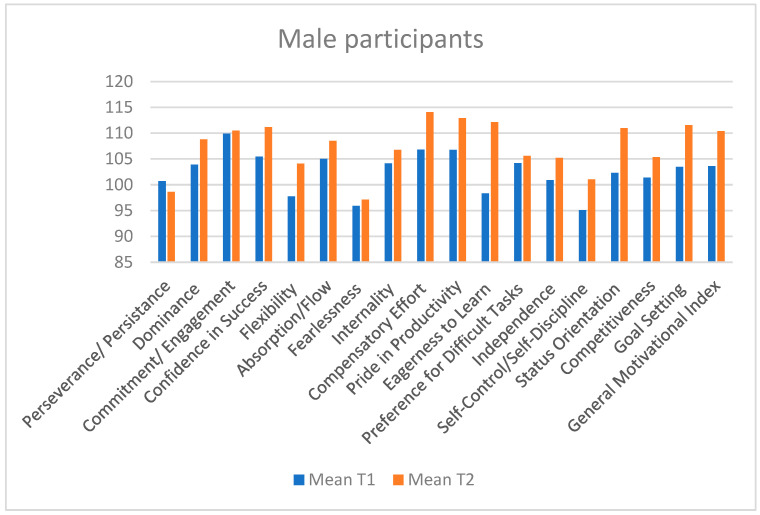
Descriptive analysis of test 1 and 2 results based on Male subjects on the overall mean score.

### 3.2. Observable Improvements After Intervention in Female Subjects (Table 8, Figure 3)

- Perseverance: 95.17 → 99.30; the increase in perseverance reflects an improvement in intrinsic motivation and mental resilience among athletes. A higher level suggests that athletes become more capable of achieving long-term goals and overcoming difficult moments in their careers.
- Dominance: 97.11 → 102.32; this increase suggests a significant improvement in self-confidence and athletes’ willingness to take an active and dominant role in competitions. Athletes become more comfortable asserting their position and making decisions under pressure.
- Goal Setting: 95.37 → 102.74; in high-performance sports, goal setting is crucial for improving results. The fact that athletes have become more efficient in establishing and pursuing their objectives has contributed to the improvement of other motivational scales, fostering a winning mindset.

The difference between the two genders may indicate that male athletes are more receptive to the implemented interventions or that the coaching strategies were more effective for them. In contrast, female athletes might require different techniques to generate significant changes in motivation and performance.

For female athletes, the Wilcoxon test (Table 8) did not reveal statistically significant changes. This suggests that the applied intervention did not have a strong enough impact on the measured psychological variables for this group.

**Table 8:** Descriptive Analysis—Female Group (Initial and Final Testing).

Indicators	N		Mean		Standard Deviation		Minimum		Maximum	
Dimensions	I	F	I	F	I	F	I	F	I	F
Perseverance/Persistence	10	8	95.17	99.297	8.413	9.207	79.68	86.33	106.28	112.93
Dominance	10	8	97.11	102.32	11.66	7.046	78.60	92.48	109.46	114.09
Commitment/Engagement	10	8	95.33	103.20	7.022	13.10	83.53	86.44	103.94	122.89
Confidence in Succes	10	8	91.60	99.602	11.18	11.01	73.59	87.56	109.90	114.09
Flexibility	10	8	98.11	95.461	6.594	9.895	84.82	77.73	106.10	109.65
Absorption/Flow	10	8	99.11	104.46	9.087	9.974	80.52	91.46	113.35	124.30
Fearlessness	10	8	93.42	93.630	19.18	11.60	62.12	75.97	124.45	113.37
Internality	10	8	103.12	105.73	7.187	8.802	86.90	93.53	113.39	118.36
Compensatory Effort	10	8	97.14	102.52	15.51	15.48	61.66	82.18	114.44	129.11
Pride in Productivity	10	8	98.48	105.49	12.43	8.462	67.95	94.23	114.26	115.51
Eagerness to Learn	10	8	95.03	100.27	8.130	11.35	81.77	81.77	106.77	116.38
Preference for Difficult Tasks	10	8	94.23	99.64	10.32	8.440	75.46	90.79	106.11	117.61
Independence	10	8	94.49	98.71	8.575	7.446	87.74	82.67	111.39	106.32
Self-Control/Self-Discipline	10	8	89.91	96.10	5.991	10.47	83.86	79.54	104.03	114.12
Status Orientation	10	8	95.67	102.70	12.08	10.22	73.32	87.29	113.98	115.25
Competitiveness	10	8	96.75	98.81	9.417	12.59	77.28	78.68	108.10	122.10
Goal Setting	10	8	95.36	102.73	13.23	12.05	66.68	89.37	112.06	121.78
General Motivational Index	10	8	94.18	101.00	6.647	8.729	78.40	88.47	100.78	114.59

**Figure 3:**
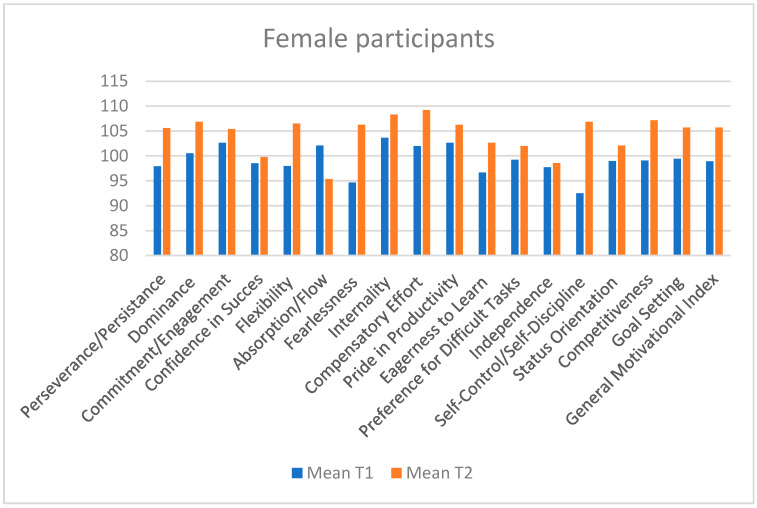
Descriptive analysis of test 1 and 2 results based on Female subjects on the overall mean score.

In synthesis, a comparison pre-test and post-test between female and male swimmers revealed that male athletes significantly improved their motivational dimensions in 8 out of 18 due to the psychological training, while the female group was much less receptive to the implemented intervention, with no significant increases at all levels.

Analyzing the values in Table 9, one may observe that most variables had a normal distribution (Shapiro–Wilk; *p* > 0.05) and met the homogeneity of variances (Levene; >0.05) assumptions. An important exception is Persistence, which does not meet the homogeneity of variances criterion that is eliminatory for ANOVA. For Persistence we applied Wilcoxon, which showed no significant difference between testing (1 and 2) and gender (Male/Female). One may observe very few exceptions of normality for Dominance, Fearlessness, Compensatory Effort for Male, T2 and Independence and General Motivational Index for Female T1.

We present in Table 10 the comparisons of the variances across the means of female and male participants.

The ANOVA table emphasize that there are significant differences between groups (Female/Male) for Commitment/Engagement (F = 10.264, *p* = 0.003), Confidence in Success (F = 11.096, *p* = 0.002), Compensatory Effort (F = 5.536, *p* = 0.025), Pride in Productivity (F = 7.160, *p* = 0.011), Status Orientation (F = 4.463, *p* = 0.042), and General Motivational Index (F = 7.348, *p* = 0.010).

## 4. Discussion

Our analysis focused on high-performance swimmers in Romania, aged between 16 and 22 years, as we observed that the critical period in athletes’ development occurs within this age range. This finding is also supported by other [17], who, based on a meta-analysis of 10 relevant articles on dropout in football, stated that “the annual weighted mean dropout rate is 23.9% across the included cohorts”. They also found that annual dropout rates remain stable between the ages of 10 and 19, with higher rates for girls (26.8%) compared to boys (21.4%). Data indicate that up to two-thirds of participants aged 7 to 18 drop out of sports each year, with attrition rates being particularly high during adolescence.

This fact was also highlighted during our study, in which four athletes withdrew. The main reasons included academic commitments and the perception of an overly demanding training environment. The analysis of AMI scores revealed significant improvements in motivation and perseverance among athletes who completed the intervention.

The six-month duration of this research was intended as an exploration of the role of psychological preparation in competitive swimming, with the objective of assessing its influence on athletes’ mental states and overall performance. Given the rigorous demands of high-performance swimming, psychological resilience is a critical factor in maintaining motivation and achieving optimal results. By implementing a structured intervention within this timeframe, we sought to evaluate its immediate impact on athletes’ engagement, mindset, and training outcomes.

Nonetheless, discussions with the participating swimmers indicate that several athletes continue to apply key elements from the intervention beyond the study period. Many reported enhanced communication with their coaches and a more positive training environment, both of which are essential for sustained performance development. These qualitative findings suggest that psychological training and emotional support may have enduring benefits, emphasizing the necessity of ongoing psychological preparation in competitive swimming.

The results of the inferential analysis showed that our intervention led to significant increases in the scores for confidence in success, compensatory effort, and goal setting, suggesting that these aspects of motivation can be influenced through specialized interventions. Our findings align with the self-determination theory, which emphasizes the importance of autonomy, competence, and social relationships in developing intrinsic motivation [18].

The analysis of Student’s *t*-tests (Table 6) highlighted statistically significant improvements in several motivational variables essential for sports performance, namely eagerness to learn (*p* = 0.035), status orientation (*p* = 0.03), and the Wilcoxon test revealed significant difference for general motivational index (*p* = 0.020).

These increases indicate greater athlete engagement in the proposed intervention plan, particularly in terms of their ability to make extra efforts to overcome difficulties and set clear and ambitious goals.

The gender differences observed in various motivational aspects related to sports performance in our study are consistent for coaches who will incorporate these findings in their training approach. However, their study revealed that female respondents perceived a higher level of achievement motivation compared to male respondents.

In a nutshell, we can assert that our findings are confirmed by similar studies Fraser-Thomas, Cote and Deakin (2008), Uzzell, Knight, Pankow and Hill (2023), which stress the role of the training patterns and coach influence within a carefully designed sport programs not just on the performing athlete, but also on his psychosocial individual development [11,19].

## 5. Conclusions

The intervention plan we applied proved to be more effective for male athletes, showing a significant increase in motivational variables essential for sports performance.

In contrast, for female athletes, the intervention did not produce statistically significant results. Although improvements were noticed in most variables, they did not reach the significance threshold necessary to be considered statistically relevant.

The initial lower values recorded by female athletes in some variables, such as perseverance and desire to learn, may explain the relatively lower impact of the intervention on this group. These initial differences could be attributed to several factors, including the possibility that female athletes may face unique psychological or social challenges that were not fully addressed in the intervention. For instance, social expectations or the influence of external factors such as family and academic pressures may have a stronger impact on the motivation of female athletes compared to their male counterparts.

This may require motivation and coaching strategies that directly target weaker aspects, such as perseverance and the eagerness to learn.

Research has shown that female athletes, particularly those in adolescent and early adulthood stages, may be more motivated and confident when they can identify with a role model who embodies traits they admire, and who reflects their personal values and aspirations [20].

It is important to note, however, that societal expectations around female athletes’ physiques often differ from those of their male counterparts. Studies have highlighted that many young female athletes express concerns about their bodies becoming too muscular, which can deter them from fully embracing certain aspects of athletic training [21]. This is particularly relevant in sports where strength and muscle development are mandatory, such as swimming, athletics, or weight training. Female athletes may feel conflicted about achieving the necessary physical strength for high performance while maintaining a traditionally feminine body image.

Emerging from our findings some practical coaching recommendations could be useful in this respect; namely, integrating coaching techniques focused on emotional self-regulation, social support, and competitive stress management on a regular basis. The constant evaluation of athletes’ progress remains essential to adjusting interventions based on individual needs and results. In the context of developing a high-performing and sustainable sports system, integrating such strategies into athlete training can ensure continuity in competitive swimming and reduce the dropout rate, thereby strengthening the future of this sport at the national and international level.

The results of this study strengthen the necessity to conceive a training process with multidimensional outcomes, covering a wide range of physical, cognitive, emotional or social variables, managed by the coach and his professional staff. In the long run, sport professionals should try to incorporate within the lesson plans more varied contents, despite the training matrices applicable to cyclic sports, often related to installing psychological barriers and dropout syndrome in practitioners. Also, coaches should create instructional situations which could lead to enhancing responsibility and commitment in view to meet the targeted objectives.

### Research Limitations

Despite the valuable insights gained, several limitations must be acknowledged. The relatively small sample size (N = 20, later reduced to 16) restricts the generalizability of the findings. Future research should expand the participant pool to enhance the reliability and applicability of the results. Additionally, the study’s follow-up period was limited to six months, providing only a short-term perspective on the effects of the intervention. A longer longitudinal analysis would be necessary to determine whether the observed improvements in motivation and communication persist over time. Furthermore, the absence of a control group limits the ability to isolate the specific effects of the psychological intervention, as comparisons with athletes who did not receive the intervention could have strengthened the study’s conclusions.

Recognizing these limitations, we view this study as an important foundation for further research. Future investigations should incorporate larger sample sizes, extended follow-up periods, and control groups to provide a more comprehensive understanding of the long-term effects of psychological interventions.

## Figures and Tables

**Table 1:** Reasons for dropout in competitive swimming.

Subject	Reason for Dropout
Subject 5	Difficulty balancing training and academic studies; age as a determining factor.
Subject 10	Lack of satisfactory results; training schedule conflicting with school schedule.
Male—Subject 4	Lack of satisfactory results; acknowledgment of physical limitations for high-performance sports.
Male—Subject 8	Prioritization of professional career; full commitment to academic studies.

**Table 2:** The annual dynamics of the number of participants.

	Year 2022		Year 2023		Year 2024	
Competition	Total Athletes	Gender/Sex Athletes	Total Athletes	Gender/Sex Athletes	Total Athletes	Gender/Sex Athletes
Children’s Pentathlon	366	M—203 F—163	396	M—216 F—180	212	M—115 F—97
Children’s National Championships	274	M—150 F—124	287	M—169 F—118	236	M—220 F—178
Cadets’ Regional	648	M—396 F—252	594	M—246 F—218	486	M—254 F—232
Cadets’ National Championships 50 m.	395	M—237 F—158	368	M—224 F—138	321	M—167 F—154
Cadets’ National Championships 25 m.	492	M—311 F—181	452	M—286 F—166	360	M—196 F—164

**Table 3:** Descriptive Analysis of the initial testing for the entire group.

Variable	N	Mean	Standard Deviation	Minimum	Maximum
Perseverance/Persistence	20	97.9310	11.95543	79.68	122.23
Dominance	20	100.5095	11.53303	78.60	123.35
Commitment/Engagement	20	102.6235	10.95837	83.53	127.26
Confidence in Success	20	98.5205	12.68912	73.59	122.47
Flexibility	20	97.9425	8.14312	84.82	113.19
Absorption/Flow	20	102.0620	8.82613	80.52	116.09
Fearlessness	20	94.6710	17.01940	62.12	125.83
Internality	20	103.6255	9.69722	85.25	123.33
Compensatory Effort	20	101.9795	14.38579	61.66	126.17
Pride in Productivity	20	102.6150	10.37771	67.95	114.26
Eagerness to Learn	20	96.6725	12.64630	68.31	122.15
Preference for Difficult Tasks	20	99.2150	14.63449	75.46	134.85
Independence	20	97.7040	10.35274	86.05	118.14
Self-Control/Self-Discipline	20	92.5060	7.65361	83.86	109.80
Status Orientation	20	98.9835	10.52308	73.32	115.25
Competitiveness	20	99.0610	12.28953	75.88	124.90
Goal Setting	20	99.4160	12.51563	66.68	118.54
General Motivational Index	20	98.9000	10.16257	78.40	126.03

**Table 4:** Descriptive Analysis of the final testing (Post-Intervention) for the entire group.

Variable	N	Mean	Standard Deviation	Minimum	Maximum
Perseverance/Persistence	16	98,9681	11,58868	82,34	119,57
Dominance	16	105.5694	9.42863	92.48	127.98
Commitment/Engagement	16	106.8600	13.05214	86.44	128.72
Confidence in Success	16	105.3850	12.99986	87.56	129.46
Flexibility	16	99.7694	9.35501	77.73	114.96
Absorption/Flow	16	106.4906	12.23130	88.73	128.40
Fearlessness	16	95.3694	13.95823	75.97	131.37
Internality	16	106.2525	9.80278	91.83	123.33
Compensatory Effort	16	108.3075	13.70806	82.18	129.11
Pride in Productivity	16	109.2075	7.24906	94.23	116.76
Eagerness to Learn	16	106.2181	13.42488	81.77	133.69
Preference for Difficult Tasks	16	102.6238	12.78648	81.21	131.02
Independence	16	101.9700	9.45006	82.67	123.21
Self-Control/Self-Discipline	16	98.5825	12.44564	79.54	128.53
Status Orientation	16	106.8288	10.09962	87.29	122.87
Competitiveness	16	102.0763	13.17615	78.68	127.70
Goal Setting	16	107.1381	13.49227	89.37	134.75
General Motivational Index	16	105.6881	11.87146	88.47	134.11

**Table 5:** Normality and Homogeneity of Variances Assumptions.

Normality Test (Shapiro–Wilk)			Homogeneity of Variances Test (Levene’s)				Normal	Homogen
Indicator	W	*p*	F	df	df2	*p*		
Perseverance/Persistence	0.96	0.269	0.017	1	34	0.9	Yes	Yes
Dominance	0.95	0.143	1.359	1	34	0.25	Yes	Yes
Commitment/Engagement	0.97	0.453	0.754	1	34	0.39	Yes	Yes
Confidence in Success	0.98	0.742	0.186	1	34	0.67	Yes	Yes
Flexibility	0.97	0.39	0.065	1	34	0.8	Yes	Yes
Absorption/Flow	0.98	0.73	1.851	1	34	0.18	Yes	Yes
Fearlessness	0.96	0.261	1.207	1	34	0.28	Yes	Yes
Internality	0.98	0.624	1.121	1	34	0.3	Yes	Yes
Compensatory Effort	0.97	0.356	0.042	1	34	0.84	Yes	Yes
Pride in Productivity	0.87	<0.001	0.501	1	34	0.48	NO	Yes
Eagerness to Learn	0.98	0.882	0.072	1	34	0.79	Yes	Yes
Preference for Difficult Tasks	0.96	0.21	0.132	1	34	0.72	Yes	Yes
Independence	0.94	0.05	0.778	1	34	0.38	Yes	Yes
Self-Control/Self-Discipline	0.96	0.224	3.015	1	34	0.09	Yes	Yes
Status Orientation	0.97	0.487	0.079	1	34	0.78	Yes	Yes
Competitiveness	0.96	0.23	0.156	1	34	0.7	Yes	Yes
Goal Setting	0.99	0.929	0.242	1	34	0.63	Yes	Yes
General Motivational Index	0.94	0.04	0.858	1	34	0.36	NO	Yes

**Table 6:** Independent Samples *t*-test.

Student’s *t*	Statistic	df	*p*	Mean Difference	SE Difference	Cohen’s d	Sig.
Perseverance/Persistence	−0.262	34	0.795	−1.037	3.96	−0.0879	No
Dominance	−1.416	34	0.166	−5.06	3.57	−0.4748	No
Commitment/Engagement	−1.059	34	0.297	−4.236	4	−0.3552	No
Confidence in Success	−1.596	34	0.12	−6.865	4.3	−0.5352	No
Flexibility	−0.626	34	0.535	−1.827	2.92	−0.21	No
Absorption/Flow	−1.262	34	0.216	−4.429	3.51	−0.4231	No
Fearlessness	−0.132	34	0.896	−0.698	5.28	−0.0444	No
Internality	−0.804	34	0.427	−2.627	3.27	−0.2696	No
Compensatory Effort	−1.339	34	0.189	−6.328	4.73	−0.4491	No
Eagerness to Learn	−2.19	34	0.035	−9.546	4.36	−0.7345	Yes
Preference for Difficult Tasks	−0.734	34	0.468	−3.409	4.65	−0.2461	No
Independence	−1.276	34	0.21	−4.266	3.34	−0.4281	No
Self-Control/Self-Discipline	−1.802	34	0.08	−6.076	3.37	−0.6044	No
Status Orientation	−2.262	34	0.03	−7.845	3.47	−0.7588	Yes
Competitiveness	−0.709	34	0.483	−3.015	4.26	−0.2376	No
Goal Setting	−1.777	34	0.085	−7.722	4.35	−0.596	No
Note. H_a_ μ_1_ ≠ μ_2_							
Wilcoxon Test for the Two Group-Level Assessments							
				Z	*p*		
Pride in Productivity				−1.864 ^b^	0.062 NO		
General Motivational Index				−2.327 ^b^	0.020 Yes		

^b^ Wilcoxon test for variables with non-normal distribution.

**Table 9:** Normality, Homogeneity of Variances tests.

Indicator	Shapiro–Wilk *p*				Levene’s Test of Equality of Error Variances			
Gender	Female		Male					
Testing	T1	T2	T1	T2	F	df1	df2	Sig.
Perseverance/Persistence	0.8	0.6	0.34	0.42	7.755	1	34	0.009
Dominance	0.1	0.11	0.12	0.02	0.029	1	34	0.865
Commitment/Engagement	0.38	0.53	0.79	0.29	0.000	1	34	0.986
Confidence in Success	0.92	0.12	0.36	0.39	0.187	1	34	0.668
Flexibility	0.18	0.73	0.21	0.91	0.686	1	34	0.413
Absorption/Flow	0.52	0.64	0.2	0.66	1.188	1	34	0.283
Fearlessness	0.96	0.94	0.83	0.01	0.013	1	34	0.910
Internality	0.23	0.42	0.35	0.73	2.002	1	34	0.166
Compensatory Effort	0.14	0.93	0.96	0.01	1.219	1	34	0.277
Pride in Productivity	0.05	0.32	0.35	0.17	3.276	1	34	0.079
Eagerness to Learn	0.76	0.88	0.96	0.45	3.475	1	34	0.071
Preference for Difficult Tasks	0.34	0.16	0.33	0.99	4.018	1	34	0.053
Independence	0.01	0.09	0.36	0.3	1.762	1	34	0.193
Self-Control/Self-Discipline	0.07	0.99	0.83	0.73	1.212	1	34	0.279
Status Orientation	0.96	0.52	0.44	0.08	1.684	1	34	0.203
Competitiveness	0.08	0.82	0.85	0.54	1.651	1	34	0.208
Goal Setting	0.45	0.45	0.31	0.75	0.042	1	34	0.839
General Motivational Index	0.03	0.8	0.31	0.43	3.803	1	34	0.059
Wilcoxon Signed-Rank Analysis Between Initial and Secondary Test Values								
					Gender			
					Male		Female	
					Z	*p*	Z	*p*
Perseverance/Persistence					−1.183 ^b^	0.237	−0.677 ^b^	0.498

^b^ Wilcoxon test for variables with non-normal distribution.

**Table 10:** ANOVA.

		Sum of Squares	df	Mean Square	F	Sig.
Perseverance/Persistence	Between Groups	69.250	1	69.250	0.504	0.483
	Within Groups	4670.488	34	137.367		
	Total	4739.738	35			
Dominance	Between Groups	399.067	1	399.067	3.678	0.064
	Within Groups	3689.202	34	108.506		
	Total	4088.269	35			
Commitment/Engagement	Between Groups	1158.608	1	1158.608	10.264	0.003
	Within Groups	3837.938	34	112.881		
	Total	4996.546	35			
Confidence in Success	Between Groups	1479.556	1	1479.556	11.096	0.002
	Within Groups	4533.508	34	133.338		
	Total	6013.065	35			
Flexibility	Between Groups	118.810	1	118.810	1.627	0.211
	Within Groups	2483.495	34	73.044		
	Total	2602.305	35			
Absorption/Flow	Between Groups	232.207	1	232.207	2.153	0.151
	Within Groups	3666.311	34	107.833		
	Total	3898.518	35			
Fearlessness	Between Groups	77.118	1	77.118	0.314	0.579
	Within Groups	8353.242	34	245.684		
	Total	8430.360	35			
Internality	Between Groups	9.191	1	9.191	0.095	0.759
	Within Groups	3280.257	34	96.478		
	Total	3289.448	35			
Compensatory Effort	Between Groups	995.087	1	995.087	5.536	0.025
	Within Groups	6111.588	34	179.753		
	Total	7106.675	35			
Pride in Productivity	Between Groups	560.269	1	560.269	7.160	0.011
	Within Groups	2660.526	34	78.251		
	Total	3220.795	35			
Eagerness to Learn	Between Groups	453.264	1	453.264	2.527	0.121
	Within Groups	6098.742	34	179.375		
	Total	6552.006	35			
Preference for Difficult Tasks	Between Groups	602.048	1	602.048	3.399	0.074
	Within Groups	6022.847	34	177.143		
	Total	6624.895	35			
Independence	Between Groups	375.197	1	375.197	4.034	0.053
	Within Groups	3162.531	34	93.016		
	Total	3537.728	35			
Self-Control/Self-Discipline	Between Groups	232.359	1	232.359	2.237	0.144
	Within Groups	3532.238	34	103.889		
	Total	3764.597	35			
Status Orientation	Between Groups	485.101	1	485.101	4.463	0.042
	Within Groups	3695.997	34	108.706		
	Total	4181.098	35			
Competitiveness	Between Groups	268.960	1	268.960	1.730	0.197
	Within Groups	5285.638	34	155.460		
	Total	5554.598	35			
Goal Setting	Between Groups	636.805	1	636.805	3.866	0.057
	Within Groups	5600.051	34	164.707		
	Total	6236.856	35			
General Motivational Index	Between Groups	797.215	1	797.215	7.348	0.010
	Within Groups	3688.624	34	108.489		
	Total	4485.839	35			

## Data Availability

The link to publicly raw datasets is https://drive.google.com/drive/folders/1NXcGOTh8hfcwi4A2LHaUov_Qjavu4ntT (accessed on 10 April 2025).

## Abbreviations

The following abbreviations are used in this manuscript:

AMI	Achievement Motivation Inventory
MZC	Mesostructure
LTAD	The Long-Term Athlete Development
G.R.O.W.	Goal, Reality, Options, and Will
SMART	Specific, Measurable, Achievable, Relevant, and Time-Bound

## Appendix A

**Table A1:** Training periodization.

A1a. Training Plan of the 2023–2024 Season							
Period/Stage	Mezostructures	Activity Location	No. of Training Days	No. of Specific Training Hours	No. of General Physical Training Hours	No. of Rest Days	No. of Competition Days
MACROSTRUCTURE I: 28 August–31 December 2023							
Basic Preparation Stage	MZC 1. 28 August–24 September	Steaua-Ghencea Base	24	72	12	4	-
Specific Preparation Stage	MZC 2. 25 September–22 October	Steaua-Ghencea Base	24	72	16	4	-
Pre-competition Stage	MZC 3. 23 October–5 November	Steaua-Ghencea Base	12	36	4	2	-
Competition Period	MZC 4. 6 November–12 November	Olympic Swimming Pool, Otopeni	3	9	1	0	4
Transition Stage	MZC 5. 13 November–26 November	Steaua-Ghencea Base	11	32	8	3	-
Basic Preparation Stage	MZC 6. 27 November–31 December	Steaua-Ghencea Base	23	62	14	14	-
MACROSTRUCTURE II: 1 January–21 April 2024							
Basic Preparation Stage	MZC 7. 1 January–21 January	Steaua-Ghencea Base	16	50	8	3	-
Specific Preparation Stage	MZC 8. 22 January–31 March	Steaua-Ghencea Base	60	180	30	10	-
Pre-competition Stage	MZC 9. 1 April–14 April	Steaua-Ghencea Base	12	36	4	2	-
Competition Period	MZC 10. 15 April–21 April	Olympic Swimming Pool, Otopeni	2	6	1	0	5
MACROSTRUCTURE III: 23 April–21 July 2024							
Transition Stage	MZC 11. 23 April–5 May 5	Steaua-Ghencea Base & Lia Manoliu Complex	17	52	8	3	-
Specific Preparation Stage	MZC 12. 6 May–30 June 30	Steaua-Ghencea Base & Lia Manoliu Complex	48	144	24	8	-
Pre-competition Stage	MZC 13. 1 July–14 July	Steaua-Ghencea Base & Lia Manoliu Complex	12	36	4	2	-
Competition Period	MZC 14. 14 July–21 July	Olympic Swimming Pool, Târgu Mureș	4	8	1	0	3
Transition Stage/Non-specific Preparation	MZC 15. 22 July–27 August	Steaua-Ghencea Base & Forban Sports Complex	17	34	12	24	-
Legend	Macrostructure—long-term training period, completed with competitions Mezostructure—medium term training period						
** A1b. Training Volume and Frequency Across Different Preparation Stages in Competitive Swimming **							
** Period/Stage **		**No. of km per Week**			**No. of Training Sessions per Week**		
Basic Preparation Stage		30–40			6–8		
Specific Preparation Stage		45–60			8–10		
Pre-competition Stage		30–40			8		
Competition Period		20–30			4–5 days of competition		
Transition Stage		45			8		
